# Visuospatial orienting in working memory is preserved in adults with ADHD symptoms

**DOI:** 10.1007/s00426-026-02411-5

**Published:** 2026-11-04

**Authors:** Luísa Superbia-Guimarães, Amy L. Atkinson, Richard J. Allen, Valérie Camos

**Affiliations:** 1 School of Psychology, University of Leeds, Leeds, UK; 2 Department of Psychology, Lancaster University, Bailrigg, UK; 3 Department of Psychology, University of Fribourg, Fribourg, Switzerland

**Keywords:** Working memory, Attention, Visuospatial orienting, Cueing, ADHD

## Abstract

People with attention-deficit/hyperactivity disorder (ADHD) are reported to have pervasive deficits in working memory (WM), yet mechanistic explanations for such deficits are lacking. Research suggests that attentional mechanisms such as reward-based prioritisation and visuospatial orienting are intact in this population (Atkinson et al., 2025; Superbia-Guimarães et al., 2022, 2023). To further examine these mechanisms, we investigated the hypotheses that deficient visuospatial orienting during encoding and maintenance are at the root of WM deficits in adults with ADHD symptoms. Eighty-four symptomatic participants (*M*_*age*_ = 28.4 years, *SD* = 4.6) and 107 controls (*M*_*age*_ = 29.4, *SD* = 4.4) had their symptoms assessed with the Adult ADHD Self-Report Scale. They each took part in one of two experiments involving an item (colour or unfamiliar shape) recognition memory task that incorporated pre- and retro-cues. Across both experiments, we found strong evidence of beneficial pre- and retro-cueing effects upon memory accuracy and reaction time (RT). However, we found no evidence for the expected group differences nor the interaction between symptom status and cueing condition. There was also no evidence for correlations between ADHD symptom scores, recognition accuracy, RT, and cueing benefits. Therefore, failure in attentional orienting is unlikely to explain deficits in WM in individuals with symptoms of ADHD.

**Supplementary Information:**

The online version contains supplementary material available at https://doi.org/10.1007/s00426-026-02411-5.

## Public significance statement

This study helps clarify why adults with ADHD symptoms often report difficulties with working memory. We tested whether problems with directing attention to important information might explain these difficulties but found that adults with ADHD symptoms use attentional cues just as effectively as those without symptoms, at least in controlled experimental settings. These results suggest that working memory challenges in ADHD are unlikely to stem from basic attentional orienting, pointing researchers and clinicians toward other possible causes.

Attention deficit/hyperactivity disorder (ADHD) is estimated to affect around 7% of children and between 2% and 7% of adults globally (Ayano et al., 2023; Salari et al., 2023; Song et al., 2021; Thomas et al., 2015). In addition to persistent symptoms of inattention and hyperactivity, ADHD is also often associated with working memory (WM) difficulties (Kofler et al., 2018, 2020; Ramos et al., 2020). WM refers to the ability to store and process a limited amount of information over short delays, in order to accomplish goal-directed behaviour (Baddeley et al., 2021; Cowan, 2017). The present work aims at elucidating one possible mechanistic explanation for the association between ADHD symptoms and WM difficulties in the adult population. Specifically, we examine whether the deficient control of visuospatial attention during information encoding and maintenance accounts for WM difficulties reported in people with ADHD symptoms.

The association between ADHD symptoms and WM abilities have long been documented in the medical literature (e.g., Martinussen et al., 2005). Some proposals even suggest that dysfunctions in WM are at the root of inattentive and hyperactive behaviours (Kofler et al., 2008), with attempts to pinpoint the defective mechanism(s) including rapid forgetting rates, defective implementation of verbal rehearsal, and central executive dysfunctions in WM (e.g., Alderson et al., 2013; Rapport et al., 2001, 2009, 2022). However, the evidence is mixed: some studies suggest that the central executive component of WM accounts for most performance variability in ADHD (Rapport et al., 2008, 2009), whereas others report broad impairments across both phonological and visuospatial WM subsystems, independent of central‑executive demands (Alderson et al., 2013); still others highlight disproportionately severe deficits in the visuospatial domain (Martinussen et al., 2005) and no deficits in the implementation of verbal rehearsal (Rapport et al., 2022).

The research outlined above has traditionally relied on neuropsychological paper-and-pencil tests, standardised computerised assessment batteries, and structural modelling techniques. These approaches are grounded in model-specific, modular conceptions of WM - most notably the multicomponent model - in which WM is conceived into separable subsystems dedicated to the processing and storage of phonological and visuospatial information, along with an attentional central executive component (Baddeley & Hitch, 1974, 1994; for recent conceptualisations of the model see Hitch et al., 2025 and Allen et al., 2026). Consequently, such studies focused largely on identifying which module within WM was presumed to underlie the observed deficits in people with ADHD.

More recently, experimental studies have adopted a more processual approach by investigating specific stages of information processing in WM. Narimoto et al. (2018), for example, investigated both the encoding and maintenance stages. They hypothesised that the lower WM performance often observed in children with ADHD may result from deficiencies in either encoding or maintenance processes. To test this, the researchers used a computerised Corsi blocks task designed to measure visuospatial WM. In this task, participants view a display containing 9 squares in an unordered spatial arrangement; they are presented with sequences of flashing squares, which they must reproduce after a delay. The authors manipulated presentation speed (fast vs. slow) and recall delay (immediate vs. delayed). The rationale was that if encoding deficiencies account for lower WM performance in children with ADHD, then slower presentation rates should compensate for these difficulties and thereby bring performance closer to that of typically developing children. In contrast, if maintenance difficulties are the primary source of impairment, then delayed recall should disproportionately hinder children with ADHD, while having minimal impact on typically developing children. The authors observed an overall and comparable benefit of slower presentation rates to both groups, and hindrance in delayed recall was only observed in the ADHD group, thus supporting the interpretation of a maintenance deficit in WM. It is worth noting, however, that the effects observed by Narimoto et al. (2018) were small and that their study was likely underpowered due to sample size limitations, thereby warranting further investigation.

Superbia-Guimarães et al. (2022) adopted a different experimental approach to test the hypothesis of encoding versus maintenance deficits in WM in children (aged 10–16 years) with ADHD. Instead of manipulating the duration of presentation and retention intervals, they manipulated the controlled orienting of attention at encoding and maintenance with endogenous spatial cues. The task involved a colour recognition paradigm in which a spatial cue (a centrally presented arrow pointing towards one location in the visual array) preceded (pre-cue) or followed (retro-cue) the encoding of a colour array. Pre-cues indicate the location of the relevant item before the presentation of the stimulus array, thereby orienting attention perceptually during encoding. Retro-cues indicate the location of the relevant item after the array has been encoded, during the retention interval of the trial, thereby orienting internal attention to the mental representation of the array. Both pre- and retro-cues robustly enhance item recognition and precision in healthy children, and in young and older adults (Griffin & Nobre, 2003; Shimi et al., 2014; Shimi & Scerif, 2022; Souza, 2016; Zheng et al., 2024) by strengthening the representation of the cued item in WM (but see Souza & Oberauer, 2016, for alternative accounts). The authors hypothesised that deficits in controlled visuospatial orienting during encoding should diminish the benefit yielded by pre-cues in comparison to typically developing children. On the other hand, deficits in the ability to orient internal attention towards the memorised array should cause smaller retro-cue benefits in the ADHD group. Contrary to expectation, beneficial pre- and retro-cue effects on recognition accuracy and reaction time (RT) were observed in both ADHD and typically developing children, with similar magnitudes between the groups. Note, however, that the study by Superbia-Guimarães et al. (2022) also had important sample size limitations, and that symptom- and age-heterogeneity was high. The authors tentatively concluded that the controlled orienting of visuospatial attention at encoding and maintenance is preserved in children and adolescents with ADHD.

A study with a more robust sample size by Atkinson et al. (2025) showed that another type of attentional mechanism in WM – reward-based prioritisation (also referred to as value-directed or value-based prioritisation) – is preserved in adults with ADHD symptoms, hence strengthening the claim that attentional processes are preserved in this population. Reward-based prioritisation refers to the ability to prioritise the maintenance of items that are deemed more important for the ongoing task. This is often captured experimentally by assigning higher or lower reward values (typically, notional points in a scoring system) to items in the memoranda, with the assumption that participants are encouraged to prioritise high-value items in detriment to low-value ones (Allen et al., 2025). Note that, reward-based prioritisation is a more controlled and strategic process and is often implemented in a sustained way throughout the experimental session, in contrast to the cue-based orienting taking place in each trial.

In line with findings in the general population (Allen et al., 2025; Allen & Ueno, 2018; Cinar et al., 2026), Atkinson et al., (2025) observed improved memory for high-value items in both ADHD-symptomatic and non-symptomatic adults, with effects being similar across the groups. The authors also found no significant correlations between the degree of inattentive and hyperactive symptoms (as measured by the Adult ADHD Self-Report Scale, Epstein et al., 2011) and the prioritisation boosts yielded. These findings are in line with those reported by Superbia-Guimarães et al. (2022), who observed no group differences between ADHD and typically developing children in the ability to use endogenous cues to prioritise items in WM. Taken together, these findings suggest that individuals with ADHD are just as capable as neurotypical individuals of strategically prioritising information in WM based on reward value, as well as effectively allocating visuospatial attention both during perceptual encoding and towards internal representations of the memorised array.

The absence of baseline group differences found in these two studies is unexpected and sharply contradicts the well-established clinical and empirical evidence of WM deficits in ADHD. Findings that fail to detect group-level differences in attentional processes in WM – either cue-based orienting or reward-based strategic prioritisation – challenge prevailing theoretical models of ADHD (e.g., Kofler et al., 2008) and raise important questions about methodological, sample-related, or task-specific factors that may obscure possible WM deficits in this population. Moreover, they highlight the need to clarify when WM impairments are detectable in experimental tasks and how mechanistic explanations for such impairments can be achieved. This study tackles two related possible explanations for ADHD-related deficits in WM, that is, that deficiencies in the control of visuospatial attention during either encoding or maintenance underlie lower WM performance in this population. We do so by building on the work of Superbia-Guimarães et al. (2022) and testing whether symptomatic young adults benefit from pre- and retro-cues in visual WM recognition tasks to the same extent as asymptomatic, healthy controls. Extending this work to symptomatic adults offers two key advantages. First, it enables a substantially larger sample (hence greater statistical power) and allows for correlational analyses between self-reported symptom scores and WM performance; in the child study that directly preceded this one, the smaller sample size precluded such analyses. Second, adults typically show a more stable pattern of symptom presentation. By contrast, the age range in Superbia-Guimarães et al. (2022; 10–16 years) corresponds to a developmental period in which symptom presentation and diagnostic status often fluctuate (Larsson et al., 2011; Norman et al., 2023). 

## Rationale and hypotheses

The rationale for this study is that deficiencies in perceptual orienting during encoding should be seen in group differences in the pre-cue benefit, whereas deficiencies in orienting attention internally during maintenance should be seen in differences in the retro-cue benefit. We examine this in recognition tasks requiring memory for colours (Experiment 1) and unfamiliar shapes (Experiment 2), for which memory accuracy served as the primary outcome measure – reflecting successful retrieval of information – with reaction time (RT) included only as a secondary, ancillary measure to support interpretation. Based on the extensive literature reporting WM deficits in people with ADHD and the literature on cueing effects in healthy adults, we hypothesised: *H1)* A main effect of ADHD symptom status upon WM performance, whereby participants with inattentive and hyperactive symptoms should have overall lower recognition accuracy compared to asymptomatic adults. *H2)* A main effect of cueing condition, whereby recognition accuracy is increased, and RTs are faster in pre- and retro-cue trials compared to baseline, neutral cue trials. *H3)* If difficulties in the controlled attentional orienting explain WM deficits in ADHD, we should observe an interaction between group and cueing condition, such that cueing effects are reduced or absent in the ADHD symptom group relative to the control group. More specifically, the reduction or absence of pre-cue effects in the ADHD group would suggest a deficit in the orienting of perceptual attention at encoding, whereas the reduction or absence of retro-cue effects would suggest deficits in the control of internal attention during maintenance. The latter outcome would extend the findings of Narimoto et al. (2018), according to which lower WM capacity in ADHD is associated with deficits in maintenance processes. Alternatively, no interaction between group and cueing would reinforce the findings of Superbia-Guimarães et al. (2022), thereby lending support to the claim that controlled attentional orienting in WM is preserved in people with symptoms of ADHD. Supplementary to hypotheses 1–4, we also hypothesised that retro- and pre-cueing effects would negatively correlate with symptoms scores, such that smaller cueing effects are associated with the presence of more symptoms.

## Experiment 1

### Method

#### Participants

Based on the sample size reported by Atkinson et al. (2025), who had 40 participants per group, and to ensure an equal number of participants in each of the six counterbalanced blocked order conditions, we aimed at a minimum size sample of 42 participants in each group (i.e., 7 participants per order per group). An a priori power analysis conducted in G-Power indicated that a minimum of 116 participants were needed across both groups to yield power of 0.95 and a small effect size of *f* = 0.15 in a 3 × 2 mixed design (condition: control, pre-cue, retro-cue; group: with ADHD symptoms, no symptoms). In anticipation of dropouts and exclusions, we collected data from 128 participants aged between 18 and 35 years, all UK residents and fluent in English.

After exclusions, 47 participants were included in the ADHD Symptoms group (*M*_age_ 28.6 years, *SD* = 4.6 years, 30 women, 17 men) and 59 in the control (no symptoms) group (*M*_age_ 29.4 years, *SD* = 4 years, 28 women, 31 men). Participants with a diagnosis and/or suspicion of autism spectrum disorder, and those who reported taking medication for ADHD symptoms were excluded from the sample. The inclusion criterion for the ADHD Symptoms group was scoring above the threshold in the Adult ADHD Self-Report Scale (ASRS, Epstein et al., 2011; Kessler et al., 2005; described in detail below), regardless of the presence of a formal medical diagnosis. The inclusion criteria for the No-Symptoms group required participants to score below the ASRS threshold and to have no medical diagnosis of ADHD, no clinical suspicion of the disorder, and not be undergoing any diagnostic investigation for ADHD.

Thirty-six participants across the sample in Experiment 1 had a formal diagnosis of ADHD but note that the group allocation was primarily determined by the ASRS classification - which reflects the current presentation of symptoms - rather than diagnostic status at the time of testing. This means that undiagnosed but symptomatic participants were allocated to the ADHD Symptoms group, and that formally diagnosed but currently asymptomatic participants were excluded from the sample. The final number of formally diagnosed participants in the ADHD-symptoms group was 16 (out of 47 participants); this low proportion of formal diagnoses in the symptomatic group is consistent with evidence indicating that many adults with clinically significant ADHD symptoms remain undiagnosed despite experiencing substantial functional difficulties (Kessler et al., 2006; Rivas-Vasquez et al., 2023; Staley et al., 2024). No participants were excluded in Experiment 1 for performing below chance level (50% correct recognition).

Participants were recruited and took part in Experiment 1 via Prolific, an online testing platform, during October and November 2025. All participants gave consent by clicking in a virtual “I accept” button before taking part in the study. The study was approved by the local Ethics Committee at the University of Leeds (approval number: PSCETHS-1148).

#### Materials and Stimuli

##### ASRS screener

Participants were screened for inattentive and hyperactive symptoms using the short version of the Adult ADHD Self-Report Scale (ASRS) (Epstein et al., 2011; Kessler et al., 2005). The short-form ASRS is a widely used screening tool with good psychometric properties, including high diagnostic accuracy, internal consistency, and test-retest reliability (Brevik et al., 2020; Kessler et al., 2005, 2007; Lewczuk et al., 2024; Matza et al., 2011), and it has been shown to perform comparably to the original 18-item version of the scale (Brevik et al., 2020; Kessler et al., 2005). The ASRS screener consists of six items assessing the frequency of symptoms experienced over the past six months, rated on a Likert scale with the following options: “Never”, “Rarely”, “Sometimes”, “Often”, and “Very Often”. In line with ethical approval, an additional response option, “Prefer not to answer”, was included. Following the scoring guidelines, participants received a score for each item if they answered “Sometimes,” “Often,” or “Very Often” on items 1–3, and “Often” or “Very Often” on items 4–6. Scores of four or higher were considered above the threshold for ADHD symptoms (i.e., symptomatic participants), whereas scores of three or lower were classified as below the threshold. A total ASRS score (possible range: 0–24) was also computed by assigning 0–4 points (“Never” = 0 to “Very Often” = 4) to each of the six items, with higher total scores indicating greater inattentive and hyperactive symptoms. Subscale scores were derived for inattention (range: 0–16, based on items 0–4) and hyperactivity (range: 0–8, based on items 5–6).

##### Working memory task

Stimuli consisted of six coloured circles measuring about 1 cm in diameter. The colours were blue (RGB: 0, 0, 225), brown (RGB: 139, 69, 19), green (RGB: 0, 255, 0), orange (RGB: 255, 165, 49), pink (RGB: 255, 105, 180), red (RGB: 255, 0, 0), violet (RGB: 148, 0, 211), and yellow (RGB: 255, 255, 0). A white square with sides approximately 1 cm in length served as the neutral cue. Four white arrows also measuring about 1 cm were used as location cues; the arrows were oriented toward the upper right, upper left, lower right, and lower left corners of the screen. A plus sign measuring about 0.5 cm was used as central fixation cross. Stimuli were presented against a grey background (RGB: 128, 128, 128). The task was implemented in PsyToolkit (Stoet, 2010, 2017).

#### Design and procedure

The task consisted of a probe recognition procedure combined with the pre- and retro-cueing paradigms (Griffin & Nobre, 2003). We used a 2 × 3 between-within design with group (ADHD symptoms, no-symptoms) and cueing condition (control, pre-cue, retro-cue). In every trial, participants were presented with a central fixation cross for 500 ms, followed by an interval of 2100 ms and the presentation of an array containing four coloured circles, with no colour repetition, for 100 ms. The location of the circles in the array was fixed to the corners of an imaginary square centred at the fixation point. Following a retention interval of 4100 ms, participants were then presented with a colour probe for recognition. Responses were given via mouse clicks on two virtual buttons saying “yes” and “no” on the bottom of the response screen. Time for response was limited to 10,000 ms from the onset of the probe to prevent participants from taking long pauses during the task. The next trial would start automatically in case of response timeout.

**Fig. 1:**
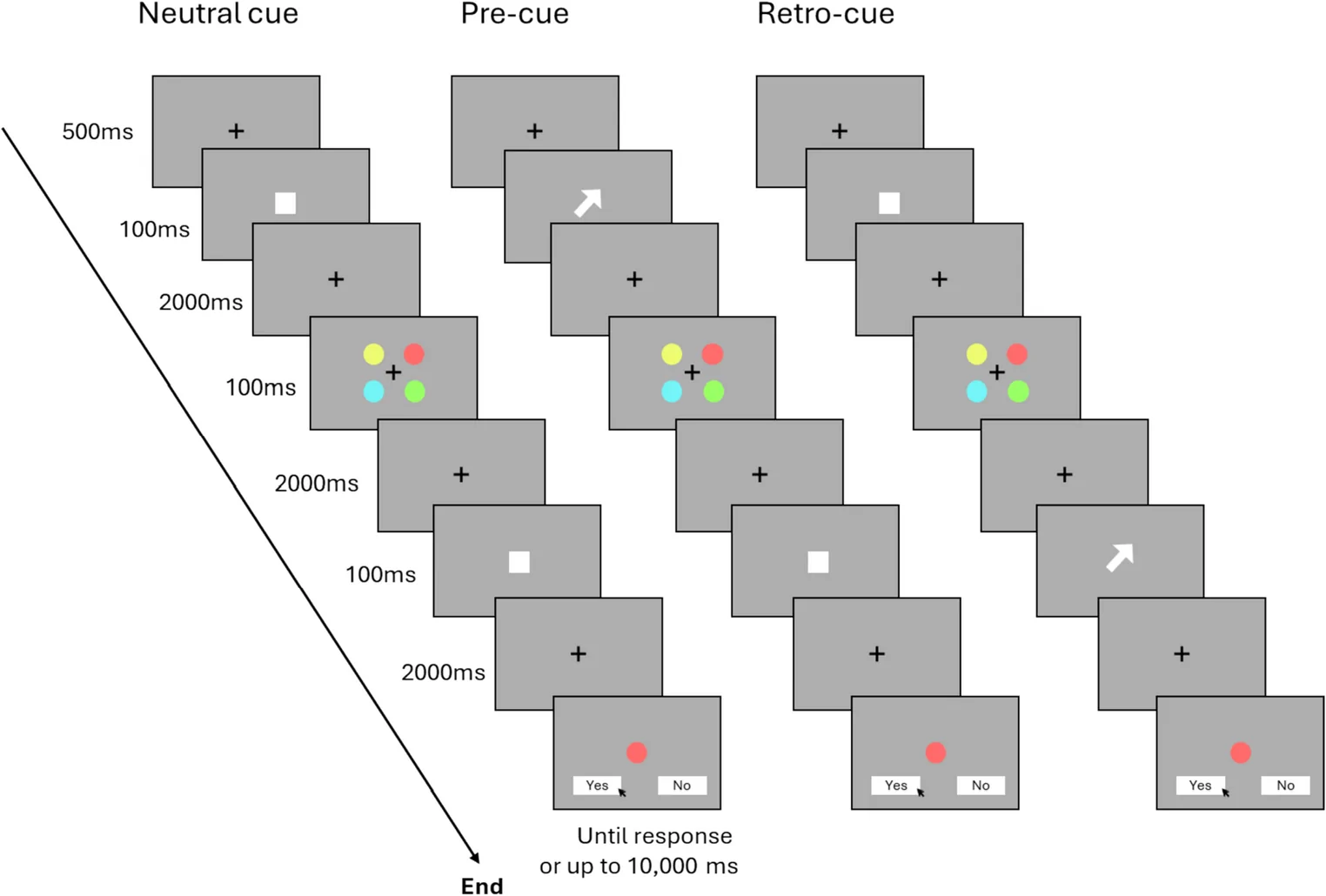
Procedure of Experiment 1. Note: The figure is not to scale. In probe-absent trials (not shown in the example), the probe colour did not appear in the stimulus array for that trial.

In the baseline condition, a neutral, non-informative cue (a white square, henceforth neutral cue) was presented at two time points during each trial: at encoding, before the presentation of the colour array, and at maintenance, during the interval between the array and the probe. In the pre-cue condition, a location cue (an arrow pointing to one of the four locations in the array) preceded the colour array, and a neutral cue was presented during the interval between array and the probe. In the retro-cue condition, a neutral cue preceded the array, and a location cue was presented during the interval between the array and the probe. The presentation time for the colour array and cues was 100 ms and the inter-stimulus interval (ISI) was 2000 ms. Figure 1 represents the details of the experimental procedure.

Each of the six colours in the stimuli pool was presented the same number of times across the four possible locations in the array, and each location was cued the same number of times across pre- and retro-cue conditions. The probed colour was present in half of the trials and absent in the other half (randomly intermixed), and the pre- and retro-cues were 100% valid in probe-present trials. Also, each colour was used an equal number of times as lures in negative probe-trials. The cueing condition was manipulated between blocks and the order of presentation of blocks was counterbalanced between participants. Each block had 24 trials that were preceded by block-specific instructions and 4 practice trials, for a total of 72 trials and 12 practice trials in the experiment. Participants were tested individually in their own PC/laptop devices via Prolific and were instructed to take the task in a quiet, isolated room. The mean study completion time was 23 min.

#### Data analysis

We used independent samples Bayesian *t*-tests to test differences in the hyperactivity, inattention and total symptoms scores between the two groups, prior to running our main analysis. Data was analysed in terms of response accuracy - our primary outcome variable – measured as correct response rates and *d’* (a measure of the discriminability between targets and lures), and RTs, measured in milliseconds. For the calculation of *d’* we used Snodgrass and Corwin’s (1988) algorithm for correcting hit and false alarm rates of 1 and 0. For the RT analysis, only trials with correct probe recognition were included, but no trimming of exceptionally rapid or slow responses was applied; this was motivated by the fact that ADHD is associated with greater RT variability (Kofler et al., 2013) and, by doing so, we would risk losing trials in which the effects of interest might arise. For example, speeded responses may reflect impulsive or prepotent responding, which is often associated with hyperactive-impulsive behaviours, whereas slower responses may indicate attentional lapses or transient task disengagement (Antonini et al., 2013; Logan et al., 1997; Tamm et al., 2012). We compared group means of response accuracy, *d’*, and RT with Bayesian mixed-ANOVAS with group (ADHD Symptoms, No Symptoms) as a between-subject factor and cueing condition (neutral, pre-cue, and retro-cue) as a within-subject factor. Finally, we calculated Bayesian correlations between the symptoms scores and WM performance (accuracy and speed measures) across cueing conditions. For all analyses, the decision criterion for accepting the alternative hypothesis was a Bayes factor (BF_10_) > 3, and the criterion for accepting the null was a BF_10_ < 0.3 (Jeon & De Boeck, 2017). The choice for Bayesian over frequentist statistics was motivated by the fact that BFs are also informative of the strength of evidence in favour or against the null hypothesis – which is not possible in the frequentist approach that only allows the acceptance of the alternative hypothesis. The analysis code, datasets, stimuli and task are available on OSF: https://osf.io/asvq3.

### Results

We found decisive evidence for group differences in the scores of inattention, hyperactivity, and total symptoms in the ASRS scale (all BFs > 3 × 10^14^), thus validating our criteria for group allocation. Table 1 presents the descriptives statistics of symptoms scores in the No Symptoms and ADHD Symptoms group.

**Table 1:** Mean inattention, hyperactivity, and total symptom scores per group in Experiment 1

	ADHD symptoms	No symptoms
Hyperactivity (Range = 0–8)	6.3 (1.3) [0.2]	2.5 (2.1) [0.3]
Inattention (Range = 0–16)	12.9 (1.9) [0.3]	5.8 (3.6) [0.4]
Total score (Range = 0–24)	19.2 (2.5) [0.4]	8.3 (4.9) [0.6]

Note. Values between parentheses represent the standard deviation and values between brackets represent the standard error of the mean

**Fig. 2:**
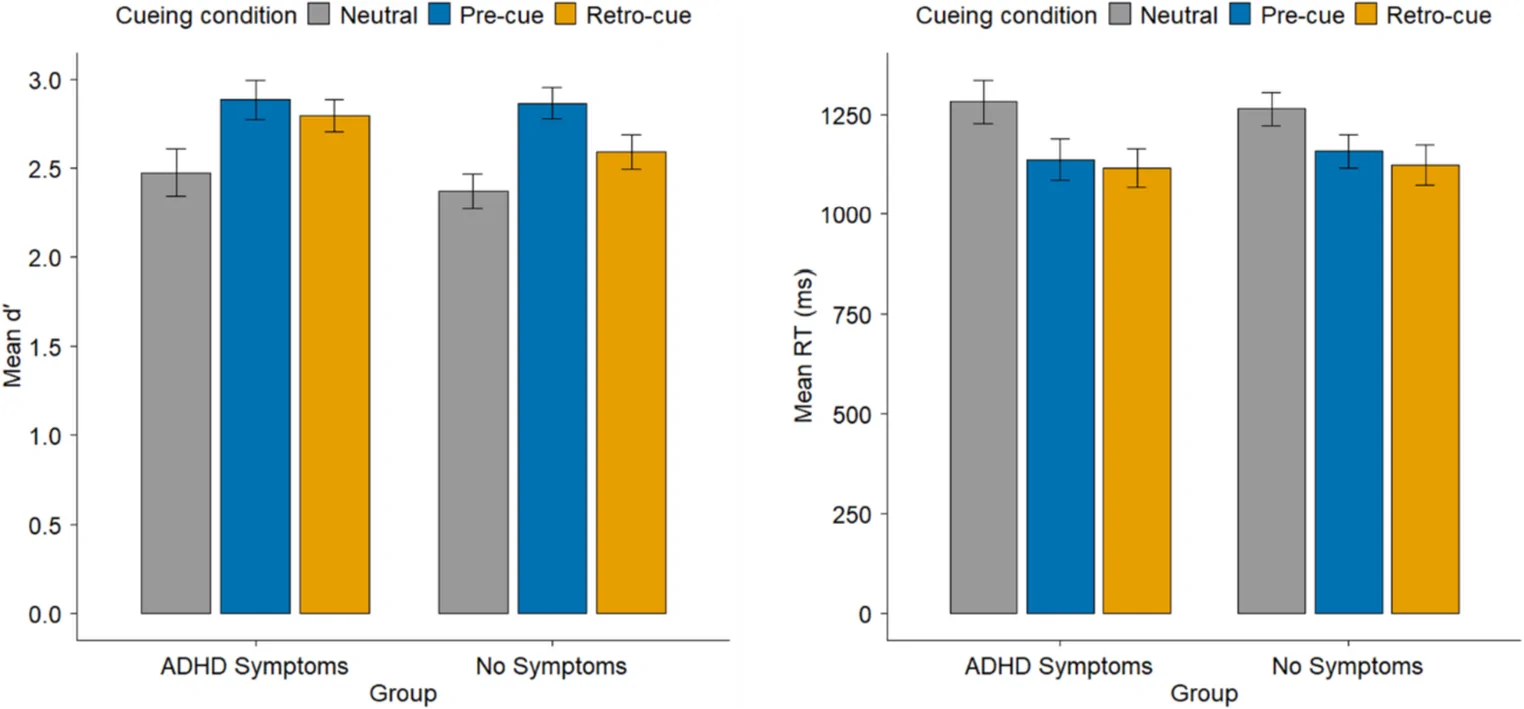
Mean d’ and reaction time by group and cueing condition (Experiment 1). Note: Error bars represent the standard error of the mean.

Missing responses due to the automatic timeout of the response screen were infrequent, with only 16 instances (0.47% of 3,384 trials; across 8 participants) in the ADHD-symptoms group and 11 instances (0.26% of 4,248 trials; across 6 participants). This amounts to a difference of only 5 missing trials between groups. Overall, missing trials constituted just 0.35% of the total dataset (7,632 trials), suggesting that missing data were negligible. Accordingly, no additional steps were considered necessary to account for missing data, and the planned accuracy and RT analyses were conducted on the available data. For simplicity, we present accuracy results only in terms of *d’*, because the response-rate analysis (available in the supplementary materials) yielded the same conclusions. Figure 2 depicts the mean *d’* (left panel) and RT (right panel) by group and cueing condition. For *d’*, the best model accounting for the data was the one including only the cueing condition (BF_10_ = 3.97 × 10^6^), with decisive evidence for the inclusion of this factor in the model (BF_incl_ = 4.01 × 10^6^). We found no evidence for a group effect (BF_incl_ = 0.34) nor for an interaction between group and cueing condition (BF_incl_ = 0.14). In fact, we found moderate evidence for the exclusion of the interaction (BF_excl_ = 7.3), and weak to moderate evidence for the exclusion of group (BF_excl_ = 2.94). Pairwise post hoc comparisons showed strong support for pre- and retro-cue benefits in recognition compared to the neutral cue condition (BF_10_ = 3.4 × 10^6^ and BF_10_ = 131, respectively), and moderate evidence for differences in performance between the pre- and retro-cue conditions, with a pre-cue advantage (BF_10_ = 3.33). According to this model, recognition memory improved with both pre- and retro-cues, but no differences were observed between those with and without ADHD symptoms. We also looked at the relationship between inattentive and hyperactive symptoms and memory accuracy from a continuous perspective. We did so by running Bayesian correlations between the scores in the ASRS scale and *d’* in each experimental condition. We found moderate evidence against a correlation between the symptoms scores and response accuracy in any of the cueing conditions (all BFs < 0.3).

Regarding the RT analysis, the best model was again the one including only cueing condition (BF_10_ = 9.02 × 10^5^), with decisive evidence for the inclusion of this factor in the model (BF_incl_ = 8.98 × 10^5^), but no evidence for the inclusion of group (BF_incl_ = 0.32) or the interaction between the two factors (BF_10_ = 0.08). In fact, we found strong evidence for the exclusion of the interaction (BF_excl_ = 11.56), and weak to moderate evidence for the exclusion of group (BF_excl_ = 3.051). Post hoc pairwise comparisons showed decisive evidence that participants were faster in pre- and retro-cue trials compared to neutral cue trials (BF_10_ < 1.30 × 10^6^ and BF_10_ = 1.57 × 10^5^, respectively), with no difference in RT between the two cueing conditions (BF_10_ = 0.2). In addition, the correlational analysis yielded moderate evidence against the presence of correlations between ASRS inattentive and hyperactive symptom scores and RTs (all BFs < 0.3). The RT analysis is congruent with the accuracy results described above: participants with and without ADHD symptoms benefitted from both pre- and retro-cues to the same extent, with no group differences in performance. A supplementary analysis comparing the gains in *d’* and RT between groups lending further support to this claim can be found in the supplementary materials.

In sum, Experiment 1 revealed strong beneficial cueing effects on both response accuracy and RT, consistent with prior findings on visuospatial orienting in WM. However, these effects were observed to the same extent for participants regardless of ADHD symptoms status, which is contrary to the hypotheses of an underlying attentional orienting deficit in WM in ADHD. This finding was reinforced by evidence against correlations between scores in the ASRS scale and measures of accuracy and RT. Moreover, the absence of a main effect of group is contrary to expectations based on literature reporting WM deficits in the ADHD population.

One shortcoming of Experiment 1 is that overall accuracy levels were high even in the baseline, neutral cue condition. Indeed, correct response rates exceeded 0.85 across all cueing conditions and groups (see Figure S1 in the supplementary materials), approaching a ceiling effect that may have prevented an interaction in which cueing effects vary by symptom status. The highly nameable nature of stimuli (colours) likely contributed to the high levels of performance, with participants possibly using verbal recoding and rehearsal to help maintain the objects. In Experiment 2, we circumvented this limitation by using a different set of stimuli to reduce nameability and thus the use of verbal rehearsal.

## Experiment 2

In Experiment 2, the colour array was replaced by an array of irregular, unfamiliar shapes. This approach was chosen because it avoids dual-task demands and attentional filtering costs associated with the implementation of other rehearsal-blocking methods, such as concurrent articulation and irrelevant speech. This is particularly important for participants with ADHD, for whom executive control and sensory filtering can be especially challenging (Friedman-Hill et al., 2010; Micoulaud-Franchi et al., 2015, 2016).

### Participants

One hundred and one participants took part in Experiment 2, none of which had taken part in Experiment 1. Sixteen participants had a medical diagnosis of ADHD in the sample. The same criteria for exclusions and group allocations were applied to the sample in Experiment 2; besides, two participants were excluded from the ADHD Symptoms group for consistently performing below chance level (i.e., 50% correct recognition). After exclusions, 37 participants (of which 3 had a medical diagnosis) were included in the ADHD Symptoms group (*M*_age_ 28.4 years, *SD* = 4.7 years, 25 women, 12 men) and 48 in the control (no symptoms) group (*M*_age_ 29.3 years, *SD* = 4.8 years, 30 women, 18 men). Data collection took place on Prolific between December 2025 and January 2026. We also ran an in-lab validation of Experiment 2 with a smaller sample (14 ADHD-symptomatic participants and 27 non-symptomatic participants) as a sanity check of our Prolific data. Group means and standard deviations in the in-lab sample closely matched that of our online sample, particularly for *d′*. Although RTs were somewhat longer in the in-lab ADHD-symptoms sample, likely reflecting differences in participants familiarity with the testing setup (e.g., use of a standard desktop mouse rather than participants’ own devices and laptop touchpads), the overall pattern of results was consistent across testing environments, reinforcing the credibility of Prolific data reported below. Full details of the in-lab methods and results are provided in the supplementary materials, and all data is available on OSF.

### Materials, stimuli, design & procedure

All experimental details were identical to Experiment 1 except that the coloured circles were replaced with irregular, unfamiliar shapes used by Hautekiet et al. (2025), and that stimuli exposure times were increased to 1000 ms. Pilot testing showed that the more complex array in Experiment 2 required saccades for full encoding, unlike the peripheral encoding of the coloured circles in Experiment 1, hence the decision to increase stimulus exposure time.

### Data analysis

The same analyses were conducted for Experiment 2.

### Results

The two groups differed in symptom presentation, as shown by higher ASRS scores of inattention, hyperactivity, and total symptoms in the ADHD symptoms group (all BFs > 1.7 × 10^9^), presented in Table 2. It should be noted that both groups exhibited scores similar to those observed in Experiment 1.

**Table 2:** Mean inattention, hyperactivity, and total symptom scores per group in Experiment 2

	ADHD symptoms	No symptoms
Hyperactivity (Range = 0–8)	6.5 (1.5) [0.2]	3 (2.2) [0.3]
Inattention (Range = 0–16)	12.5 (1.7) [0.3]	5.7 (3.2) [0.5]
Total score (Range = 0–24)	19.0 (2.3) [0.4]	8.7 (4.7) [0.7]

Note. Values between parentheses represent the standard deviation and values between brackets represent the standard error of the mean

There were 14 missing responses (0.50% of 2,808 trials; across 5 participants) in the ADHD-symptoms group and 20 missing responses (0.58% of 3,456 trials; across 7 participants). Overall, missing trials constituted 0.54% of the total dataset (6,264 trials), suggesting that missing data were again negligible in Experiment 2. To ensure that our aim of increasing task difficulty was effective, we compared memory accuracy in each cueing condition across Experiments 1 and 2 with Bayesian independent samples t-tests. This analysis yielded decisive evidence for an effect of experiment, with lower memory accuracy in Experiment 2 (all BF_10_ > 206), hence confirming that the use of irregular shapes made the task more challenging. This is likely due to a combination of factors such as increased item complexity and inter-item similarity, and a reduction in stimulus nameability and opportunity to use verbal rehearsal.

**Fig. 3:**
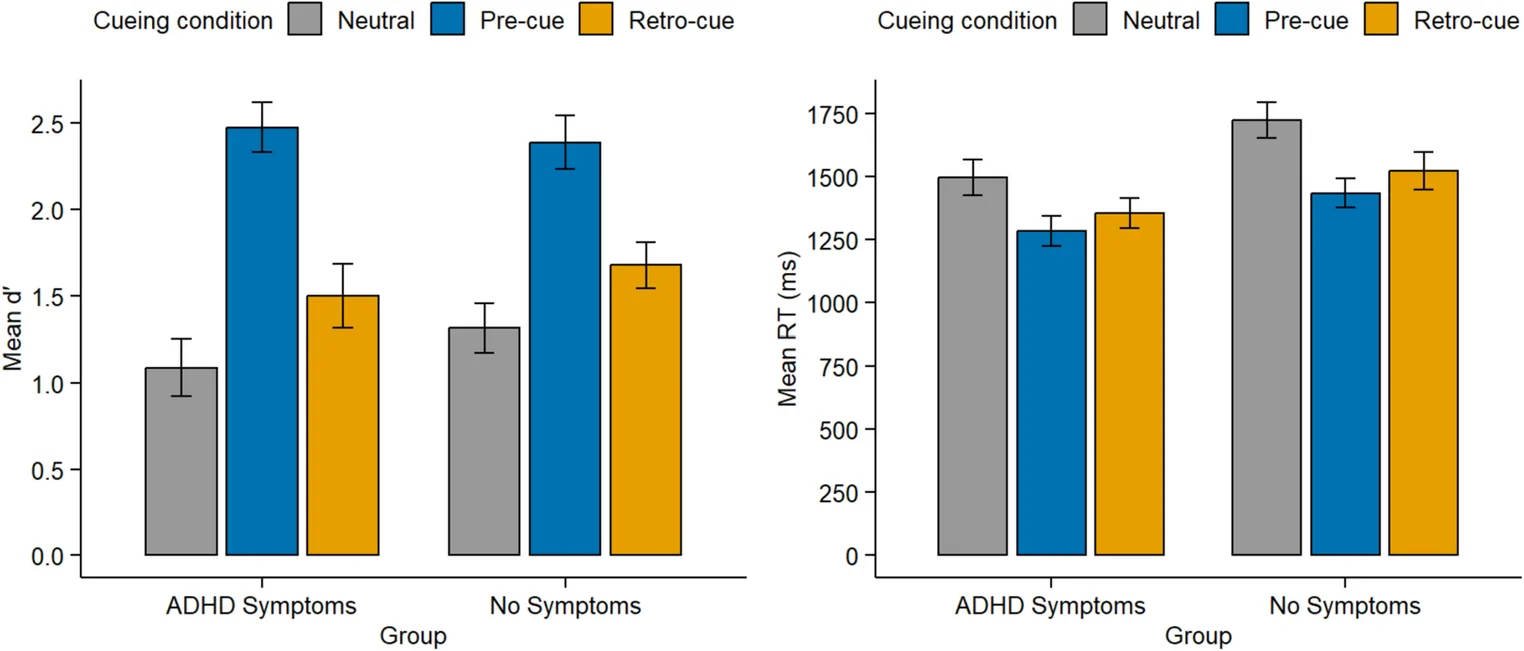
Mean d’ and reaction time by group and cueing condition (Experiment 2). Note: Error bars represent the standard error of the mean.

Figure 3 shows the mean *d’* and RT per group and condition in Experiment 2. The results of Experiment 2 are overall consistent with those of Experiment 1. For the *d’* analysis, the best model accounting for the data was the one including only the cueing condition (BF_10_ = 1.08 × 10^7^, BF_incl_ = 1.12 × 10^17^). We found moderate evidence for the exclusion of group (BF_excl_ = 3.45) and its interaction with cueing condition (BF_excl_ = 5.74). Post hoc pairwise comparisons showed decisive evidence for pre- and retro-cueing advantages compared to the neutral cue condition (BF_10_ = 2.4 × 10^12^ and BF_10_ = 45.7, respectively). There was also decisive evidence for higher *d’* in pre- than in retro-cued trials (BF_10_ = 1.19 × 10^8^), in congruence with Experiment 1, though the evidence was only moderate in that experiment. According to this model, recognition memory improved with both pre- and retro-cues, with greater improvement in pre-cue trials, but no difference between participants with and without ADHD symptoms both in terms of overall performance and in the size of cueing effects.

For the RT analysis, the best model was again the one including only cueing condition (BF_10_ = 1.71 × 10^4^, BF_incl_ = 6.8 × 10^3^), with weak evidence for the inclusion of group (BF_incl_ = 2.57) and no evidence for its interaction with cueing condition (BF_incl_ = 0.10). In fact, the latter corresponds to strong evidence for the exclusion of the interaction from the model (i.e., BF_excl_ = 10.14). Thus, although participants in the ADHD-symptoms group showed numerically faster RTs overall (Fig. 3, right panel), this difference was not meaningfully modulated by the cueing condition.

To test whether pre- and retro-cue trials differed in their effects on RT, we conducted Bayesian post hoc pairwise comparisons. These analyses provided strong evidence that RTs were faster for both pre-cue and retro-cue trials relative to neutral cue trials (BF₁₀ = 7.67 × 10³ and BF₁₀ = 15.75, respectively), with no evidence for a difference in RT between pre- and retro-cue trials (BF₁₀ = 0.59) despite a numerical trend toward slightly faster RTs in pre-cue trials. These results are aligned with evidence showing comparable speed advantages for both pre- and retro-cues (Griffin & Nobre, 2003; Lepsien & Nobre, 2006; Nobre et al., 2004).

The analysis of the cueing benefits upon the *d’* and RT was congruent with the main results and can be found in the supplementary materials. Finally, in line with the results above, we found no conclusive evidence supporting correlations between ASRS inattention, hyperactivity, or total symptom scores and either *d′* or RT measures (all BFs < 1).

In sum, using more complex stimuli that reduced opportunities for verbal rehearsal led to lower memory accuracy in Experiment 2. Crucially, however, the overall pattern of results replicated that of Experiment 1, suggesting that the absence of group differences cannot be attributed to ceiling-level performance resulting from the high nameability of the stimuli used in Experiment 1. Hence, the results of Experiment 2 reinforce the finding that both pre- and retro-cues reliably enhance recognition memory, independent of participants’ levels of inattentive or hyperactive symptoms. Note however that our manipulation in Experiment 2 did not increase memory load per se, in that the set size remained at four items – within the usual adult WM capacity range (Cowan, 2010) and in line with previous studies tapping into visuospatial orienting and prioritisation in ADHD (Atkinson et al., 2025; Superbia-Guimarães et al., 2022). It is unclear whether the same pattern would be observed at larger set sizes exceeding storage capacity.

Finally, the greater pre-cue advantage observed in Experiment 2 is aligned with previous results in the literature (e.g., Myers et al., 2015), although several studies have suggested that retro-cue benefits can be of comparable magnitude to pre-cue benefits (Griffin & Nobre, 2003; Nobre et al., 2004; Lepsien & Nobre, 2006). The pattern we observed is consistent with the notion that pre-cues can provide a greater benefit than retro-cues because they allow an anticipatory shift of attention to relevant stimuli at encoding (hence reducing the load of information entering WM), whereas retro-cues modulate access to information that has already been encoded into WM (Murray et al., 2013; Myers et al., 2015). Because differential pre- and retro-cueing effects were not the focus of the current work, we will refrain from discussing this finding in length. For the purposes of this study, we stress the fact that pre- and retro-cue advantages were comparable across the two groups.

## Discussion

This study examined the hypothesis of localised deficits in visuospatial attentional orienting during the encoding and maintenance stages of WM in adults with ADHD symptoms. In two experiments using a classic cueing paradigm, we did not find evidence of overall lower memory accuracy in symptomatic participants, nor evidence of smaller cueing benefits in this group. We also found no meaningful effects of symptoms status upon cueing benefits in RT. These results are further corroborated by no evidence for correlations between symptoms ratings in the ASRS scale and memory performance. In sum, the benefits yielded by pre- and retro-cues were similar across both groups in both experiments, both for accuracy and RT. In other words, participants with ADHD symptoms were equally adept as non-symptomatic participants in voluntarily orienting perceptual attention to relevant locations and in orienting internal attention to mental representations in WM. Moreover, despite the large and robust group differences in clinical symptoms as shown by the ASRS scores, memory performance in ADHD-symptomatic participants was no lower than in individuals without ADHD symptoms, even in the baseline neutral-cue condition. Although surprising from the point of view of the extensive literature showing WM deficits in ADHD (e.g., Kofler et al., 2018, 2020; Ramos et al., 2020), the absence of differences in baseline memory accuracy is not unprecedented (e.g., Atkinson et al., 2025; Superbia-Guimarães et al., 2022, 2023).

Our results contradict the hypothesis of an overall lower WM accuracy in symptomatic participants (*H1*). They also lend no support to the hypothesis of deficits in the voluntary control of visuospatial attention during encoding and maintenance (*H2*), thus reinforcing findings reported by Superbia-Guimarães et al. (2022) with a younger group (aged 10–16 years). In the next sections, we will discuss the absence of group differences in more detail in light of the extant literature on attentional orienting, and we speculate on the role of executive control in individuals with inattentive and hyperactive symptoms.

### Visuospatial orienting

Our results contribute to a growing body of evidence showing no or very little evidence of deficient attentional orienting in WM and in perceptual tasks in the ADHD population. In the study that was the immediate precursor to this one, we found that children and adolescents (aged 10–16 years) with diagnosed ADHD benefitted from pre- and retro-cues to the same extent as age-matched typically developing peers, both in accuracy and RT measures (Superbia-Guimarães et al., 2022). Moreover, baseline performance in neutral cue trials was not different between the two groups, in line with our current findings. The current findings with adults closely fit those developmental outcomes, and offer a twofold interpretation: first, it provides direct evidence of comparable WM accuracy between people with ADHD symptoms and typically developing individuals; second, it offers evidence of preserved visuospatial orienting in both perception and memory in people with ADHD symptoms in two different age groups.

The second interpretation is partly corroborated by perceptual studies conducted in children (Caldani et al., 2020; Huang-Pollock & Nigg, 2003) and adults (Epstein et al., 1997; Roberts et al., 2018). In children, Huang-Pollock and Nigg’s (2003) meta-analysis of 14 studies concluded that ADHD is not characterised by significant visuospatial orienting dysfunction. More recently, an eye-tracking study conducted by Caldani et al. (2020), using a modified Posner task to measure children’s oculomotor response latencies during overt shifts of attention (i.e., looking at a target appearing on the left or right side of the display), provided a more nuanced perspective on those earlier findings. In this study, attentional orienting was manipulated using exogenous cues (i.e., peripheral luminance changes on either side of the display that automatically capture attention) and endogenous cues (i.e., central arrows, like in our current study). Trials were either validly cued (i.e., cue congruent with target location), invalidly cued (i.e., cue incongruent with target location), or neutrally cued (baseline condition). No group differences in response latencies were observed in neutral-cue or valid-cue trials, for either exogenous or endogenous cueing. However, a group difference emerged in the invalid endogenous cue condition, in which children with ADHD showed slower orienting responses, whereas latencies were comparable in the invalid exogenous cue condition. This pattern suggests that children with ADHD exhibit (1) intact perceptual orienting toward targets (i.e., in neutral-cue trials), (2) preserved sensitivity to automatic/peripheral attentional capture (i.e., in valid exogenous cue trials), (3) efficient perceptual reorienting following automatic attentional shifts (i.e., in invalid exogenous cue trials), and (4) comparable voluntary orienting and anticipation (i.e., in valid endogenous cue trials). The observed impairment therefore appears to concern disengaging voluntary attention from a misleading endogenous cue (i.e., in invalid endogenous cue trials).

In adults, Roberts et al. (2018) investigated the perceptual effects of voluntary and involuntary attention using an orientation discrimination task. Although voluntary attention significantly improved performance at relevant locations and impaired performance at irrelevant locations, the magnitude of these orienting effects did not differ between ADHD-symptomatic and non-symptomatic groups. These findings contrasted with those of one of the first studies to demonstrate differences between adults with and without ADHD on an objective measure of attention. Specifically, Epstein et al. (1997) reported longer delays in adults with ADHD when attention was misdirected by cues presented in the right visual field and had to be shifted to a target presented in the left visual field, although the two groups did not differ in mean RTs overall. The authors concluded that the response pattern exhibited by adults with ADHD reflected a disengagement deficit.

Overall, findings in both children and adults tend to support the preservation of visuospatial orienting in ADHD, with only limited evidence for disengagement difficulties in the ADHD population. However, these studies involved perceptual tasks, and evidence for preserved orienting benefits in WM remains scarce, with Superbia-Guimarães et al. (2022) representing, to our knowledge, the only previous report. The current study contributes to this emerging body of evidence. Nevertheless, our study did not include invalid endogenous cues, namely those associated with slower orienting latencies in Caldani et al. (2020) and Epstein et al. (1997). Consequently, we cannot determine whether difficulties in disengaging voluntary attention from previously attended locations during encoding also extend to memory retention processes. Such a cascading effect appears plausible, and future WM studies should therefore incorporate invalid cues into their design.

### Executive attention

Deficient executive control is another central component of classic models of ADHD to account for the symptomatology reported in ADHD (e.g., Barkley, 1997). One possible explanation for the absence of baseline group differences and cueing effects across groups in the present study is that the task (recognition coupled with visuospatial cueing) did not strongly engage executive processes. However, this interpretation cannot stand for other existing evidence showing no group differences. For instance, Superbia-Guimarães et al. (2023) employed a complex span task – a task well recognised for relying on executive control – that requires participants to remember a sequence of items (e.g., letters for later recall) while performing a secondary task during the intervals between each item (e.g., judging whether a line fits the gap between two dots during the interval between the presentation of each letter in the sequence). No group differences were observed in recall performance in the baseline (low load) condition for which the cognitive load of the secondary task was titrated for each participant. Moreover, recall of both ADHD and typically developing participants was equally hindered when the attentional constraints imposed by the high-load condition was increased. Congruently, another study by Weigard and Huang-Pollock (2017) show that cognitive load manipulations impose similar constraints to memory performance in ADHD and typically developing children in a Corsi blocks task. These findings tentatively suggest that the ability to coordinate the use of attention between processing and maintenance – a core executive function – is preserved in ADHD. In other words, the capacity to flexibly switch or share attention between concurrent demands during a WM task to maintain memory items in WM appears intact in this population.

Relatedly, current evidence suggests that the implementation of another maintenance mechanism, verbal rehearsal – an operation that places relatively low demands on executive control for adults – also does not seem to be dysfunctional in ADHD. We did not observe group differences in our Experiment 2, where any possibility of rehearsal was minimised by the use of irregular, difficult-to-name shapes. Rapport et al. (2022) investigated the hypothesis of a rehearsal deficit in ADHD more directly by imposing articulatory suppression (a manipulation that prevents rehearsal) during a word recall task with lists of 2, 4, and 6 words; if verbal rehearsal were a deficient process in ADHD, the cost of articulatory suppression should be disproportionately larger on the typically developing group. Although they observed a main effect of group in memory recall (in contrast to our findings) whereby ADHD group recalled fewer words in general, they did not observe the expected interaction between group and the articulatory suppression manipulation. In other words, preventing participants to rehearse had the same detrimental effects in both groups, suggesting that this mechanism is preserved in this population.

Besides failing to observe the expected group by experimental manipulation interactions, the studies cited above share several characteristics: set sizes/list lengths were kept within WM capacity (at span level in Superbia-Guimarães et al., 2023, and up to six words in Rapport et al., 2010^1^), stimuli were relatively simple and one-dimensional (e.g., letter or word series, or locations in a spatial grid), and response demands placed relatively little strain on executive control. Evidence from tasks involving higher ecological demands and more complex stimuli offer a more nuanced picture. Yang et al. (2017), using a following-instructions paradigm, reported marked group differences, suggesting that ADHD-related impairments may become more apparent in naturalistic tasks. In their study, children aged 6–11 years were presented with sequences of verbal instructions involving object-action associations (e.g., “touch the pen, pick up the eraser, then spin the ruler”). Both presentation and recall format were manipulated: instructions were either spoken or demonstrated via video, and recall was either verbal (i.e., by verbally repeating the sequence of commands) or enacted (i.e., by executing the actions). Children with ADHD performed more poorly than typically developing children across all task conditions. However, enacted recall benefited both groups to a similar extent, again failing to reveal the expected interaction.

Although enacted recall arguably adds the complexity of motoric control at retrieval, evidence from dual-task paradigms indicates that it may not, in fact, impose greater executive demands than verbal recall (Yang et al., 2014, 2016). Thus, the benefit of enacted recall observed in ADHD may reflect a relatively low-cost strategy that remains available despite broader task difficulty and clinical impairments observed in this population. Yang et al. (2017) argue that such benefits stem from automatic processes that are independent of the effortful strategic control that may be hindered in ADHD. It should be noted, however, that this possibility sits somewhat at odds with the absence of ADHD-related deficits in strategic prioritisation in a computerised WM task reported by Atkinson et al. (2025), hence warranting the need of further investigation.

### Intentional maintenance and disengagement

A useful framework for interpreting the present null results is provided by Shipstead et al. (2016). Two controlled processes – intentional maintenance and intentional disengagement – are central to their account of executive attention. In this framework, intentional maintenance refers to the active retention of goal-relevant information in the face of distraction or interference, whereas intentional disengagement refers to the ability to suppress or remove irrelevant, outdated representations from active processing. The former is reflected in psychometric tests of WM, which require the active maintenance of information, and the latter in tests of fluid intelligence, which require the generation of novel solutions to problems (Engle, 2018).

Importantly, our task does not fully engage both dimensions of Shipstead et al.’s (2016) framework. Firstly, although it clearly places demands on intentional maintenance (i.e., maintaining the colour or cued item array until test time), these demands are constrained to brief, discrete trials that are relatively transient and self-contained, and involve minimal concurrent distraction. Regarding the second dimension of Shipstead et al.’s framework, our task requires only a minimal degree of intentional disengagement, for example, when orienting attention to retro-cued items at the expense of uncued representations that had been encoded into WM, or when moving from one trial to the next. These demands are comparatively modest and occur within brief, repeated, discrete events that minimise proactive interference from previous trials. Both intentional maintenance and disengagement are likely to be more challenging in unfolding and continuous event streams that are often intrinsic to more real-life tasks. The controlled nature of our task might have limited the emergence of group differences in both intentional maintenance and disengagement, and particularly the latter. Indeed, considering that participants voluntarily enrol to take part in the study online, one could reasonably assume that they intentionally make an effort to disengage from competing thoughts in order to participate in the experiment.

Paradigms that more fully tax intentional disengagement, particularly in continuous, interference-rich contexts, may be better suited to detecting ADHD-related WM deficits. For example, complex span and n-back tasks impose greater demands on disengagement by requiring continuous updating and the removal of no-longer-relevant information from WM. Possible ways to tap into disengagement within the current paradigm involve including either invalid cues or double retro-cues in the experimental design, although we have reservations about the former option. In tasks involving memory for one-dimensional stimuli such as ours (colours in Experiment 1; shapes in Experiment 2), invalid location cues would require participants to maintain item–location bindings throughout pre-cue and retro-cue blocks – unlike in the neutral-cue condition, where no locations in the array are cued. This would increase memory load on cued trials and introduce an additional difference between conditions beyond attentional orienting, the mechanism we aimed to isolate. A more suitable extension, consistent with our paradigm, would be the use of double retro-cues (e.g., Guo et al., 2024; Ueno & Allen, 2025), in which either the same item is cued twice during the trial or two different items are cued sequentially, with only the second tested. The latter case provides a manipulation of disengagement, as participants must abandon the first cued representation to benefit from the second cue. Future work should investigate these cueing variations as a way to test disengagement more directly.

### Study limitations

One important consideration is that our ADHD-symptoms group included a relatively low proportion of formally diagnosed participants (16 out of 47 in Experiment 1, and 3 out of 37 in Experiment 2), raising the possibility that group differences may emerge in a more clinically characterised sample. We used the ASRS screener, a self-report measure of inattentive and hyperactive symptoms, and defined group membership based on its classification threshold rather than through formal clinical interviews and/or the presence of a previous medical diagnosis. Although we acknowledge this as a limitation, we note that the absence of the expected group interactions is not unique to the present study and has also been reported in samples of people with formal diagnoses. For example, Superbia-Guimarães et al. (2022, 2023) similarly failed to observe the anticipated group differences in children with confirmed ADHD diagnoses. In those studies, diagnoses had been made by a specialist child psychiatrist, and most participants were receiving pharmacological treatment for clinically significant symptoms. Consistent with standard practice in the field, participants were tested after a 24-hour suspension of medication to ensure that ADHD symptoms were not masked during assessment. The work by Weigard and Huang-Pollock (2017) and Rapport et al. (2022), discussed above, provide additional examples of studies using stricter participant selection – including parent and teacher symptom reports across multiple clinical tools, clinical interviews, and medical referrals – in which the experimental manipulations produced the same effects across groups. Although some of these results should be interpreted cautiously given the relatively small sample sizes (namely in Rapport et al., 2022 and Superbia-Guimarães et al., 2022, 2023), the overall findings suggest that the lack of expected group differences and interaction in our current study cannot be readily attributed to our questionnaire-based group classification alone. Instead, they may point to a broader disconnect between clinically validated measures of ADHD symptomatology and performance on tasks isolating specific WM components under highly controlled laboratory conditions.

## Conclusion

Our findings suggest that visuospatial orienting in people with ADHD symptoms appears to be preserved in well-controlled WM tasks involving set-size demands that do not exceed normal capacity (i.e., between 3 and 5 items, Cowan, 2010). More broadly, across different experimental paradigms, the emerging pattern of findings provides little consistent evidence that core attentional control mechanisms in WM are impaired in people with ADHD symptoms under well-controlled conditions. Such control mechanisms include the orienting of visuospatial attention, as manipulated by cueing tasks like in the present study and in Superbia-Guimarães et al. (2022), reward-based prioritisation of items in WM (Atkinson et al., 2025), and switching attention between storage and processing demands in WM (Superbia-Guimarães et al., 2023). This raises the provocative possibility that WM function may be largely preserved in inattentive and hyperactive individuals in such settings; it also indicates a mismatch between laboratory measures and the behavioural symptoms of inattention and hyperactivity observed in clinical contexts. Real-world WM difficulties in symptomatic ADHD may emerge from the interaction of multiple factors not fully captured in laboratory tasks. These include the ability to disengage from irrelevant mental representations, such as outdated information or distracting, unrelated thoughts. More optimistically, the current results also point to the possibility that these individuals may greatly benefit from increased structure and control in real-life contexts that engage WM, such as educational and occupational settings. Further research is needed to bridge the gap between experimental findings and the real-world WM challenges that are experienced by people with behavioural symptoms of inattention and hyperactivity.

## Supplementary Information

Below is the link to the electronic supplementary material.

Supplementary Material 1

## Data Availability

Materials and data are available at OSF: https://osf.io/asvq3.

## Footnotes

1 But note that Weigard and Huang-Pollock (2017) used sequences of 2–9 spatial locations, hence including set sizes that exceeded capacity for their 8-12-year-old participants. For comparison, the average young adult capacity in the Corsi Blocks task is around seven items (Farrell Pagulayan et al., 2006).
