# Supplementary material for: Visuospatial orienting in working memory is preserved in adults with ADHD symptoms
Source: Psychol Res. 2026 Nov 4;90(6):198. doi: 10.1007/s00426-026-02411-5 (PMC13736375; doi:10.1007/s00426-026-02411-5)
Supplement: Supplementary file 1 [file 426_2026_2411_MOESM1_ESM.pdf]

Supplementary Materials for: Visuospatial Orienting in Working Memory is Preserved in Adults with ADHD Symptoms

Experiment 1

Correct Response Rates

The analysis of correct response rates yielded the same conclusions as the analysis of the *d'*. The strongest model was the one including only cueing condition ($BF_{10} = 1.36 \times 10^6$). We found moderate evidence for the exclusion of the factor group ($BF_{excl} = 3.5$) and its interaction with cueing condition ($BF_{excl} = 7.09$).

Figure S1

Correct Response Rates in Experiment 1

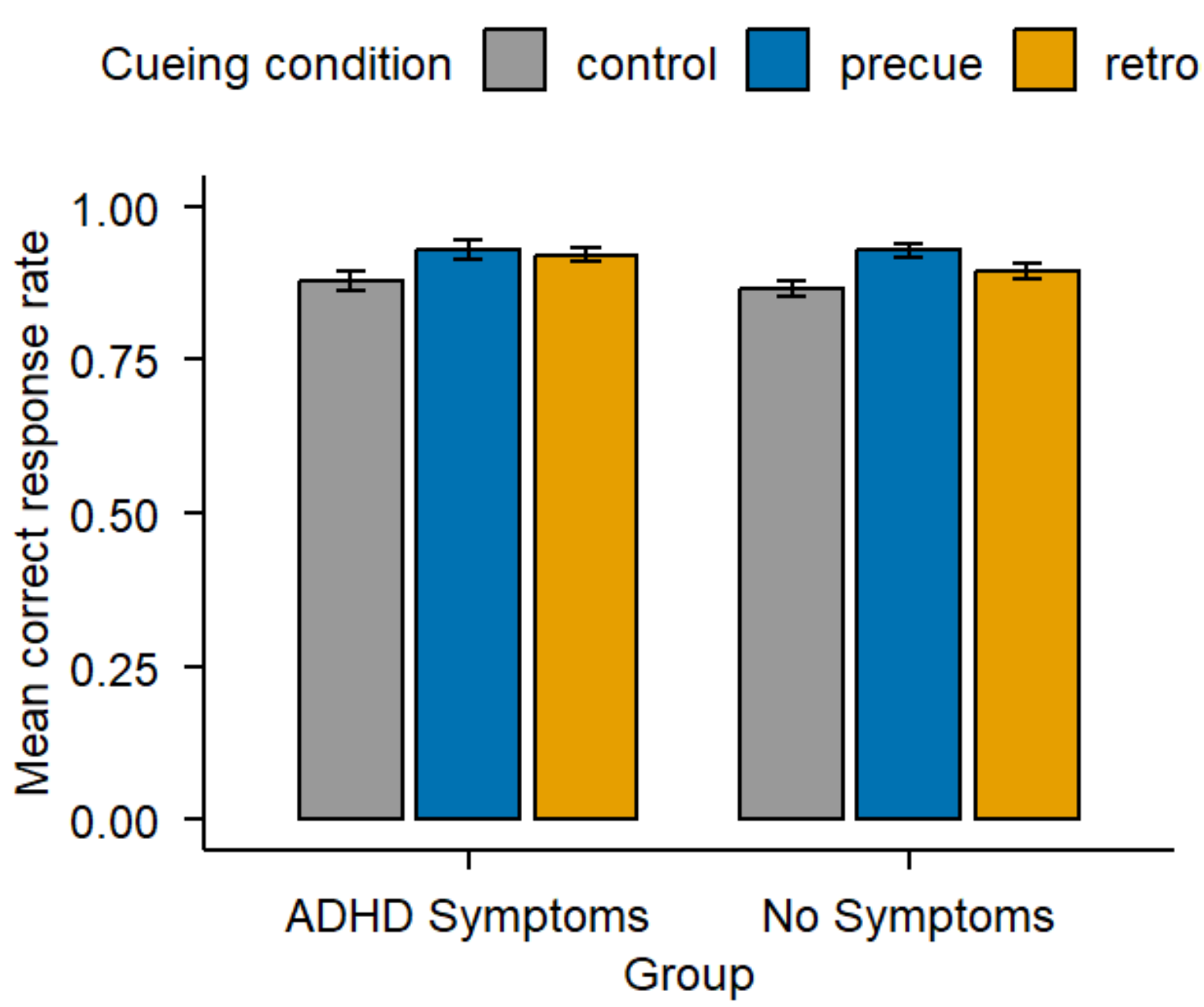

Note. Error bars represent the standard error of the mean.

Analysis of Cueing Benefits

In this analysis, we compared the gains yielded by pre- and retro-cues in terms of memory recognition and speed between the two groups of participants. The cueing benefit in accuracy was calculated by subtracting the average *d'* in the baseline, neutral cue trials from the average *d'* in pre- and retro-cue trials. For the RT analysis, the cueing benefit was calculated by subtracting the average RT of correct responses in neutral cue trials from pre- and retro-cue trials. These differences – henceforth "cueing benefits" – were then compared with Bayesian 2x2 ANOVAS contrasting benefit type (pre-cue, retro-cue) and group (no symptoms, ADHD symptoms).

A cueing benefit for the *d'* corresponds to positive values representing a gain in recognition performance yielded by the cues. The mean pre-cue benefit in the *d'* was 0.50 (*SD* = 0.68) in the No-Symptoms group and 0.40 (*SD* = 0.79) in the ADHD Symptoms group. The mean retro-cue benefit was 0.22 (*SD* = 0.73) in the No Symptoms group and 0.32 (*SD* = 0.65) in the ADHD Symptoms group. The Bayesian ANOVA showed moderate evidence against a group effect (BF_{excl} = 4.67) and against an interaction between group and benefit type (BF_{excl} = 3.65). Also, there was no evidence - albeit marginally – for a difference between the benefit type (BF_{incl} = 2.9).

For the RT, the average pre-cue benefit was -106 ms (*SD* = 202) in the No Symptoms group and -145 ms (*SD* = 205) in the ADHD Symptoms group. The average retro-cue benefit was -140 ms (*SD* = 297) in the No Symptoms group and -165 ms (*SD* = 234) in the ADHD symptoms group. Note that, for RT, cueing benefits correspond to negative values representing faster responses, i.e., a gain in speed yielded by the cues. The Bayesian ANOVA showed moderate evidence for the exclusion of benefit type (BF_{excl} = 5.36) and group (BF_{excl} = 4.97), and

strong evidence for the exclusion of the interaction with cue type from the model (BF_{excl} = 26.07).

In sum, the benefits were comparable between participants with and without ADHD symptoms both in terms of memory recognition and speed, with no differences between the two cue types.

Figure S2

Cueing Benefits in Experiment 1

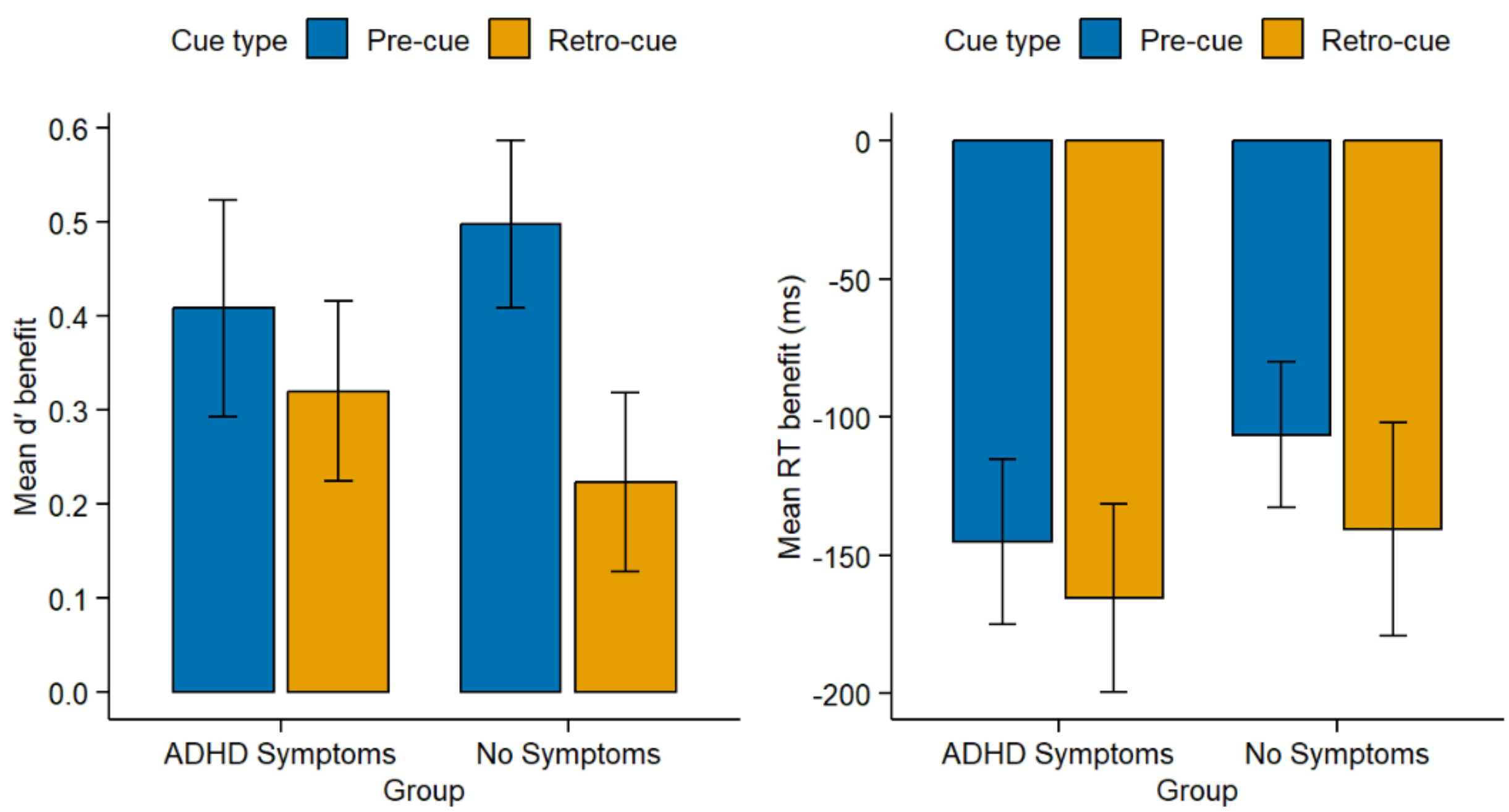

Note. Note that negative RT values (right panel) represent faster responses. Error bars represent the standard error of the mean.

Experiment 2

Correct Response Rates

The analysis of correct response rates yielded the same conclusions as the analysis of the *d'*. The strongest model was the one including only cueing condition (BF_{10} = 11.79). We found moderate evidence for the exclusion of the factor group (BF_{excl} = 3.6) and its interaction with cueing condition (BF_{excl} = 4.51).

Figure S3

Correct Response Rates in Experiment 2

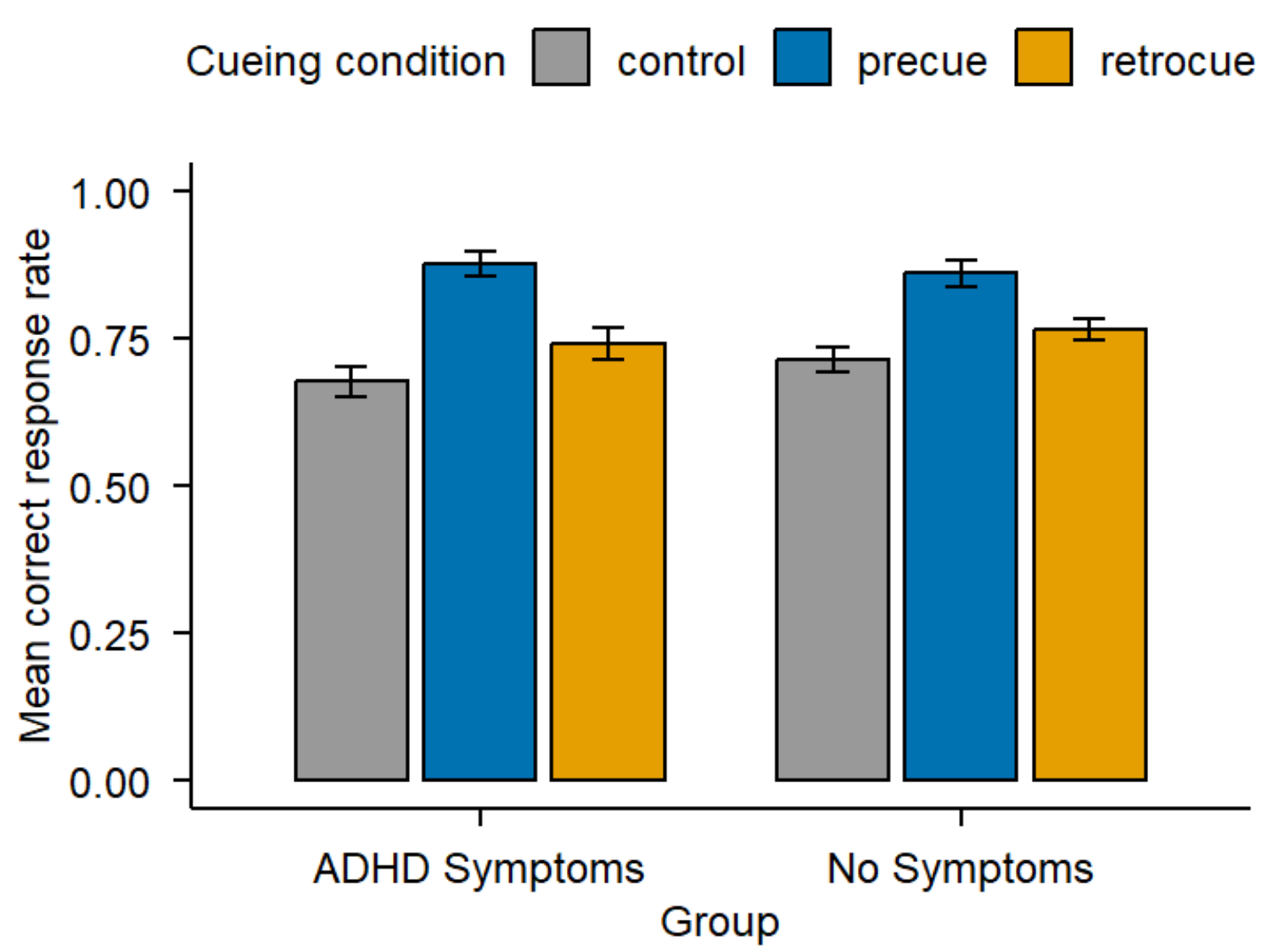

Note. Error bars represent the standard error of the mean.

Analysis of Cueing Benefits

In Experiment 2, the mean pre-cue benefit in *d'* was 1.07 (*SD* = 1.16) in the No Symptoms group and 1.39 (*SD* = 1.14) in the ADHD Symptoms group. The mean retro-cue benefit was 0.36 (*SD* = 0.93) in the No Symptoms group and 0.41 (*SD* = 1.05) in the ADHD Symptoms group. The Bayesian ANOVA showed decisive evidence for a benefit-type effect ($BF_{incl} = 1.008 \times 10^8$), with larger benefits yielded by pre- than retro-cues as shown by pairwise post hoc comparisons ($BF_{10} = 1.18 \times 10^8$). There was no evidence for a group effect (BF_{incl} = 0.40), nor its interaction with benefit type (BF_{incl} = 0.40).

The average retro-cueing benefit upon RT was -289 ms (SD = 378) in the No Symptoms group and -213 ms (*SD* = 553) in the ADHD Symptoms group. The average pre-cueing benefit was -201 ms (*SD* = 469) in the No Symptoms group and -142 ms (*SD* = 529) in the ADHD symptoms group. The Bayesian ANOVA showed no evidence for a difference between neither cue type (BF_{incl} = 0.77) nor group (BF_{incl} = 0.38), and moderate evidence against their interaction (BF_{excl} = 4.45). The results observed in Experiment 1 were replicated in Experiment 2 with slightly larger benefits to both *d'* and RT, likely due to the fact that baseline performance was lower in the second experiment, in which we purposefully used more complex and harder-to-name stimuli to minimize the use of verbal rehearsal.

Figure S4

Cueing Benefits in Experiment 2

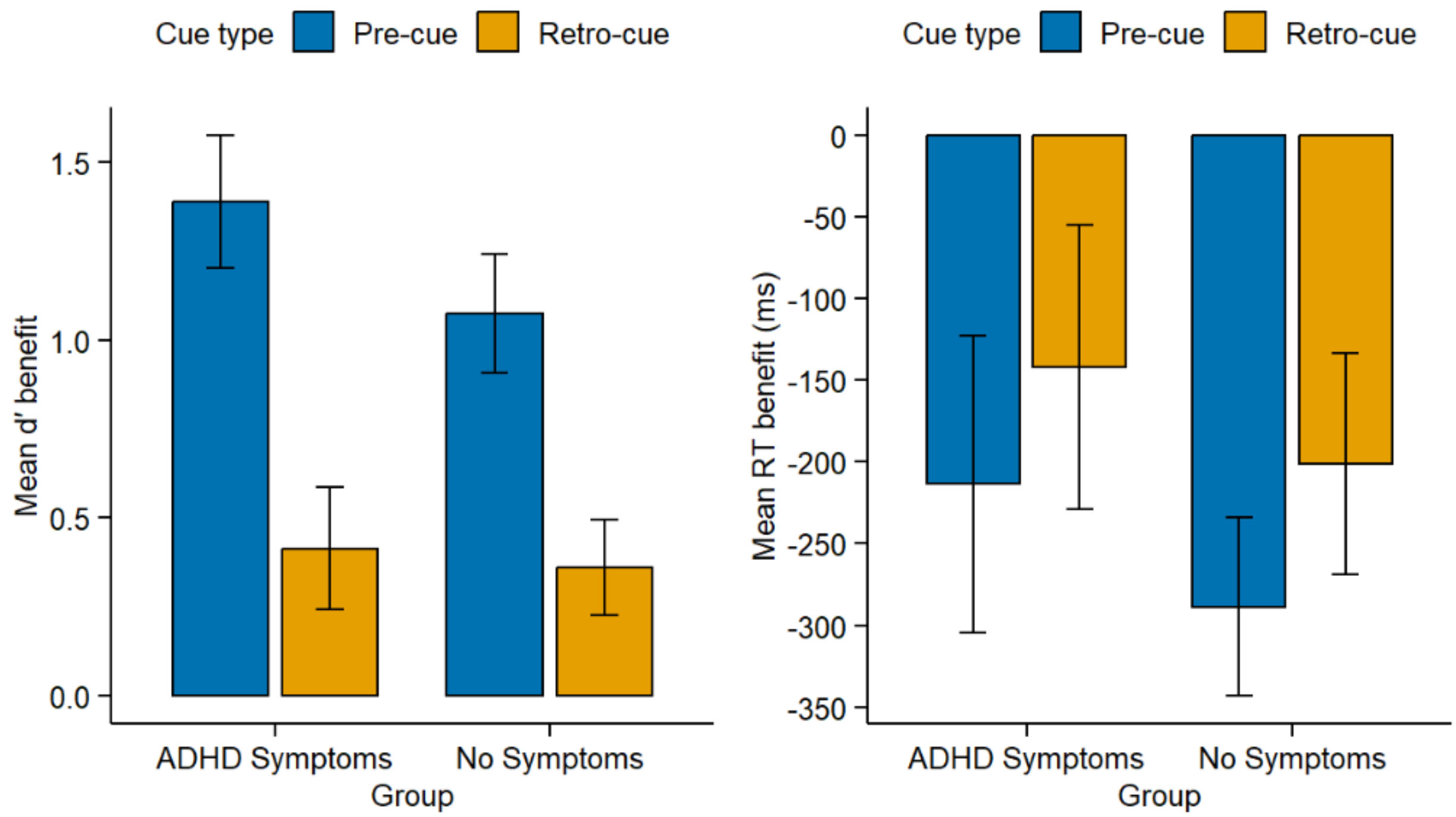

Note. Note that negative RT values (right panel) represent faster responses. Error bars represent the standard error of the mean

Robustness Check of Online Data

To evaluate the robustness of the online findings, we conducted an in-lab validation of Experiment 2 using a smaller sample. This served as a sanity check of the data collected via Prolific.

Method

The procedure was identical to that of Experiment 2, except that participants completed the study in the laboratory. Participants were either awarded course credit (if enrolled in the

School of Psychology, University of Leeds) or entered into a prize draw for participation. They were seated approximately 60 cm from a desktop computer monitor. The experimenter remained in the testing room while participants read the information sheet and provided informed consent.

Participants

Participants were recruited from the student population at the University of Leeds. The same group allocation criteria used in Experiment 2 were applied. The ADHD-symptoms group included 14 participants (M_{age} = 21 years, *SD* = 2.4, 12 women, 2 men), of which 6 had a medical diagnosis, and the no-symptoms group included 27 participants (M_{age} = 20.3 years, *SD* = 1.3, 20 women, 7 men).

Results

The group averages for symptoms scores in the Adult ADHD Self-Report Scale (ASRS are reported in table S1; averages for the d' are reported in Table S2.

Table S1

Mean of Inattention, Hyperactivity, and Total Symptom Scores per Group in Experiment 2 (In-Lab data)

	ADHD symptoms	No symptoms
Hyperactivity (Range = 0-8)	5.9 (2.0) [0.5]	3.7 (1.9) [0.4]
Inattention (Range = 0-16)	13.1 (2.2) [0.6]	6.7 (2.5) [0.5]
Total score (Range = 0-24)	19.1 (2.9) [0.8]	10.4 (3.2) [0.6]

Note. (standard deviation) and [standard error of the mean].

Table S2

Average Recognition Accuracy (d') and RT (ms) in Experiment 2 (In-Lab data)

	Accuracy (d')		RT (ms)	
	ADHD symptoms	No symptoms	ADHD symptoms	No symptoms
Neutral cue	1.31 (0.93) [0.26]	1.32 (0.82) [0.16]	1909 (417) [112]	1777 (449) [86]
Pre-cue	2.98 (0.54) [0.22]	2.42 (1.11) [0.14]	1363 (259) [69]	1427 (413) [79]
Retro-cue	1.84 (0.75) [0.20]	1.72 (1.00) [0.19]	1573 (438 [117]	1477 (429) [83]

Note. (standard deviation) and [standard error of the mean].
